# Complete genome sequence of marine filamentous bacterium, *Saprospira grandis* strain WH^T^

**DOI:** 10.1128/MRA.00441-23

**Published:** 2023-08-17

**Authors:** Wooi Liang Heng, Nyok-Sean Lau, Go Furusawa

**Affiliations:** 1 Centre for Chemical Biology, Universiti Sains Malaysia, Bayan Lepas, Penang, Malaysia; University of Southern California, Los Angeles, California, USA

**Keywords:** predator, marine microbiology, CFB

## Abstract

Here, we report the complete genome sequence of a type strain of the genus *Saprospira*, *Saprospira grandis* strain WH^T^. The genome consists of one circular chromosome and plasmid comprising 4,250,550 bp and 53,161 bp with GC content of 46.6% and 46.8%, respectively.

## ANNOUNCEMENT

*Saprospira grandis*, a Gram-negative, marine filamentous, and gliding motile bacterium (1), is the only species in the genus *Saprospira*, which is also known as a predator against bacteria, such as *Vibrio* spp. (1, 2). During predation, the vibrios were captured by extracellular sticky materials of *Saprospira* in a process called ixotrophy (3). Although draft genomes of two strains of *S. grandis*, namely, strain Lewin and strain Sa g1, were sequenced (4, 5), the genome of the type strain, WH^T^ (ATCC 23119 = JCM 21750 = LMG 10407), has not been sequenced yet.

Strain WH^T^ was obtained from the Belgian Coordinated Collections of Microorganisms (BCCM, Gent, Belgium). For DNA extraction, the strain was cultured by a high-nutrient artificial seawater broth (1-HASWM; 1% tryptone, 2.4% artificial sea salt, pH 7.6) for a day at 30°C with shaking at 200 rpm (6). The DNA was extracted using the SDS-proteinase K method (7), and the DNA was further purified with AMPure XP beads (Beckman Coulter, USA). The same extracted DNA was used for Oxford Nanopore and Illumina sequencing. The library for Oxford Nanopore sequencing was prepared using native genomic DNA barcoding with a ligation sequencing kit, SQK-LSK109. The gDNA library was loaded on the R9.4 flow cell and sequenced using the PromethION. The Nanopore sequencing data in the fast5 file were basecalled and then filtered to retain clean reads (Q ≥7) using GUPPY v5.0.16. A total of 145,504 clean reads totaled up to 147.85 million bp with N_50_ of 14,507 were obtained, and the sequencing depth is 340×. For Illumina pair-end sequencing, the sequencing library was prepared using NEBNext Ultra II DNA Library Prep Kit following the manufacturer’s protocol. The library was then sequenced on the Illumina NovaSeq 6000 platform with 2 × 150 bp chemistry. The reads were filtered with fastp v0.23.2 to remove low-quality bases and adapter sequences. The 8.94 million clean filtered reads were obtained, resulting in a sequencing depth of 310×. The clean reads from Oxford Nanopore and Illumina pair-end sequencing were used for hybrid assembly using Unicycler v0.4.9 (8). Hybrid assembly produced two complete circular contigs corresponding to a circular chromosome of 4,250,550 bp and a circular plasmid of 53,161 bp determined using PlasFlow v1.1 (9). The chromosome and plasmid have GC content of 46.6% and 46.8%, respectively. The assembled genome was annotated with NCBI Prokaryotic Genome Annotation Pipeline v6.4 (10). Default parameters were used for all software for assembly and annotation. A total of 3,386 genes were identified in the genome, including 3,310 protein-encoding genes, 60 RNA genes, and 16 pseudogenes. Functional annotation was performed using BlastKOALA against the KEGG database (11). The WH^T^ genome lacked complete genes for synthesizing essential amino acids, similar to the genome of *S. grandis* str Lewin (4). By omitting amino acids from the synthetic medium, WH^T^ required all essential amino acids except lysine for growth (12), suggesting that the bacterial predatory nature of the genus *Saprospira* is crucial for obtaining macromolecules for growth. However, the predator-prey relationship of strain WH^T^ still remains unclear (2).

## Data Availability

Whole genome sequencing of *S. grandis* strain WH^T^ was deposited in DDBJ/EMBL/GenBank under the assembly accession number CP110854.1 (chromosome) and CP110855.1 (plasmid unnamed), BioProject with the accession number PRJNA899791, and BioSample with accession number SAMN31669021. The RefSeq accession number for *S. grandis* strain WH^T^ whole-genome sequence is GCF_027594745. The filtered reads from Oxford Nanopore sequencing and Illumina pair-end sequencing are deposited on the SRA database under accession numbers SRR23239776 and SRR23239775, respectively.
